# Investigation of direct counting and sizing of DNA fragments in flow applying an improved data analysis and correction method

**DOI:** 10.1016/j.bdq.2019.100083

**Published:** 2019-03-16

**Authors:** Martin Hussels, Susanne Engel, Nicole Bock

**Affiliations:** Physikalisch-Technische Bundesanstalt, Abbestr. 2-12, 10587 Berlin, Germany

**Keywords:** Molecular quantification, Metrology, Enumeration based quantification, DNA-copy concentration, Flow cytometric counting, dPCR

## Abstract

Direct detection of single stained DNA fragments in flow is a very sensitive method for nucleic acid detection which does not need any amplification process. We have developed an instrument for direct counting and sizing of single DNA fragments (single or double stranded DNA) in flow with integrated sample volume measurement for concentration determination. As the method is a potential reference method for DNA quantification, processes affecting the measurement uncertainty are of major interest. Additionally, comparison of this method to the orthogonal method of digital PCR is useful with the restriction of low specificity of the direct detection method. In this study, we analysed raw detector signals and the sizing performance for target identification and the effect of coincidence detection concerning concentration measurements. We present data of purified artificial DNA samples measured with the home-built setup. Main emphasis was to develop an improved data analysis method to gain insight into and carefully correct for coincident detection of DNA fragments and for estimation of the amount of fragment dimers.

## 1 Introduction

Molecular methods, like real-time polymerase chain reaction (qPCR) are of increasing importance to diagnose infectious diseases e.g. tuberculosis (TB), HIV or special pathogens like Zika and Dengue. For therapeutic monitoring qPCR as a quantitative method offers the potential for a rapid and objective diagnosis, alternative to microbial culture tests. As qPCR becomes more and more relevant for clinical applications, robust mechanisms for quality control and quality assurance, including metrological controls, are required. For this purpose, higher-order methods for absolute quantitation of DNA concentration are necessary. In a recent international comparison (CCQM P154) two methods for absolute quantification of DNA copy numbers were compared to validate their potential use as higher-order methods [1]. Both methods are based on enumeration (SI unit 1) and thereby do not require calibration. One method is digital PCR, which is based on counting the number of positive reactions for a large set of parallel amplification reactions using small volume partitions. For more detailed information on dPCR see [2]. The other method is direct counting of stained DNA fragments using a modified flow cytometer, which is very sensitive and therefore does not require amplification.

The first flow cytometer optimized for detection of stained DNA fragments was already reported in 1993 where fragments down to 10 kbp have been detected [3], [4]. Further development and optimization e.g. better single photon detectors and longer dwell times lead to detection of DNA fragments down to 125 bp over the years [5], [6], [7]. Besides this detection and sizing of small fragments the sizing of large DNA fragments was extended to 500 kbp by enhanced data evaluation [7], [8]. The DNA fragment sizing is based on intercalating dyes, which show a constant ratio of base pairs per dye molecule. Consequently, the fluorescence of a single DNA fragment is proportional to the fragment length. However, the motivation was to develop a technology to measure DNA fragment size finger prints comparable to gel electrophoresis. In comparison to gel electrophoresis this method needs much less material and the sizing range from 10^2^ bp to 5 × 10^5^ bp is much larger than for conventional gel electrophoresis. For such large fragments pulsed gel electrophoresis would be necessary which needs up to 24 h for one run. Although this method has significant benefits in comparison to gel electrophoresis and quite compact and easy to handle instruments have been developed [7], it was never commercialised and somehow vanished after 2004.

One aspect, which was not studied in the first period of development of the single fragment detection is the possibility of quantification of the DNA amount or DNA fragment concentration by counting of the fragments. This offers a method to detect and quantify trace amounts of DNA fragments without amplification processes like PCR [9]. Beyond the very good sensitivity this method can be used to quantify concentration without the need of reference material by measuring the sample volume simultaneously. Although the method is excellent in identifying and counting DNA fragments (single or double stranded) of different length, it must be noted, however, that it is not specific in respect to the sequence of the detected DNA. Therefore, it cannot be used to distinguish fragments of same length but different sequence. Additionally, it is not suitable to study mixtures of ss- and dsDNA because of different fluorescence enhancement factors of these. For applications as a higher-order measurement method this means that accurate quantification with lowest measurement as well as identification uncertainty can be achieved only with artificial pure materials [9], [10], e.g. to assign conventional true measurement values to certified reference materials or other calibrators.

In this study, we applied flow cytometric detection of plasmid DNA, which was also used in an interlaboratory study on dPCR. We have set up an instrument with excellent linear dependence of the detected signal on the DNA fragment length and a sample delivery system with accurate volume measurement in the μL range. Additionally, we developed an improved data analysis method to identify coincident detection of DNA fragments and fragment dimers. As coincidences are inherent for flow cytometric counting and fragment dimers or multimers may be intrinsic for certain samples, the improved data analysis is helpful to reduce measurement uncertainty in concentration determination.

## 2 Materials and methods

### 2.1 Dedicated flow cytometer

The home-built setup for detection of single stained DNA fragments in flow uses a 488 nm Ar+ laser for fluorescence excitation and detects fluorescence between 500 nm and 550 nm (yellow green). The fluidics consists of a 10 L reservoir, which is connected subsequently to a flow sensor (Sensirion AG), a 100 nm pore size filter (Merk Millipore, Germany), and a standard flow cell for optical detection (Sysmex Partec GmbH) with a 200 μm × 350 μm channel. The outlet of the flow cell is connected to a 10 L waist container. The sheath flow is pressure driven at typical pressures of 260 mbar to 320 mbar controlled using a precision pressure control valve. The resulting sheath flow rates are 20 μL/min to 30 *μ*L/min, which is more than 250 times smaller than typical flow rates of conventional flow cytometers. We implemented a capillary with 150 *μ*m inner diameter and a length of approximately 30 cm as flow resistance element between the flow cell and the waste container to gain such low flow rates.

The sample delivery is realised using a high precision translational stage in combination with a syringe. The syringe used for experiments (1710 LT, Hamilton Bonaduz AG) with 100*μ*L volume was calibrated using the calibration method described in ref. [11]. The calibration factor was determined to (1.681 ± 0.012) μL per 1 mm piston travel of the syringe. The syringe is connected to the flow cell using a fused silica capillary (ID= 150 *μ*m, VWR International GmbH) extending directly into the sheath flow, which consists of ultra-pure water. This way, the sample does not have contact to metal or plastic parts of the flow cell, which may cause unknown adsorption losses of DNA on their surfaces. For fused silica, the adsorption can be controlled by the pH of the sample solution [12].

**Fig. 1:**
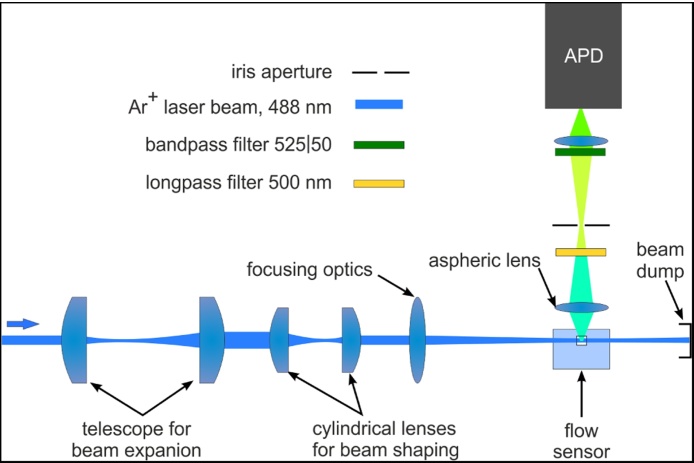
Optical setup for detection of single stained DNA fragments. The laser beam is expanded by a telescope consisting of two spherical lenses and then shaped to an ellipse by a second telescope consisting of two cylindrical lenses. The shaped beam is focused into the flow channel of a conventional flow cell where it excites the stained DNA fragments. The fluorescence signal is collected in sideward direction by an aspheric lens and focused on an iris aperture acting as spatial filter. The light passing the spatial filter is focused on a single photon counting avalanche photo diode (APD) for detection. For spectral selection a 500 nm longpass filter and a 525 nm bandpass filter with 50 nm bandwidth are inserted in the detection pathway.

Fig. 1 shows the optics of the setup developed for DNA fragment detection. The laser beam is expanded using a telescope consisting of two spherical lenses and then shaped to an elliptic form using a telescope of cylindrical lenses. This elliptical beam is then focused into the flow channel of the flow cell using a 6.3× NA 0.02 objective (Melles Griot GmbH, Germany). Due to its elliptical shape, the focus is tight in *z* direction (∼8 μm) and wider in the x-y plane (∼40 μm). This minimises the dependence of the resulting signal on the x-y position of the sample stream.

The fluorescence signal of the sample is collected with the help of an aspheric lens (*f* = 4.5 mm NA 0.56, Newport Corporation, USA), passes a 500 nm long pass filter (LOT-QuantumDesign GmbH, Germany) and is then focused on an iris aperture, which is used as a spatial filter to control the detection volume. After passing the spatial filter the fluorescence signal passes a 525|50 nm band-pass filter (LOT-QuantumDesign GmbH, Germany) and is focussed on a single photon counting APD (COUNT-10B, Laser Components GmbH) for detection.

The photon counts of the APD are acquired with a counter timer board (NI PXI-6602, National Instruments Corporation, USA), which is controlled by a self-developed software based on LabView. During measurement, the number of detected photons for adjustable time bins (typically 0.1 ms) is acquired continuously for a selectable time range giving a time trace of the signal intensity detected by the APD. Acquisition of the complete time trace gains the opportunity to apply different settings for data analysis.

### 2.2 Data analysis

The acquired time traces are analysed applying a conventional trigger algorithm for peak detection using a separate also LabView based software. The algorithm searches for data points above the threshold value given by the user. If a data point is above the threshold the algorithm evaluates all following data points until the value falls below the threshold again. Within this data evaluation the peak area is measured as sum of the data points, the peak width is measured as the number of data points and the peak height is measured as the maximum value. These three parameters are stored together with the time stamp of the first data point of the peak, but to suppress noise only peaks with a width of more than 3 data points are stored. Additionally, base line correction and photon count rate correction can be applied on the data. The resulting data is stored in FCS 3.0 format for following analysis using standard flow cytometry software.

The photon count rate correction is necessary to correct for the photon count losses of the single photon counting APD caused by the dead time *t*_D_ after each photon detection. This detector dead time leads to a decreasing detection efficiency at increasing count rates and a theoretical rate limit of 1/*t*_D_. For known detector dead time the measured count rate *C*_m_ can be corrected by:

(1) $$C=C_{m}\frac{1}{1-\left(t_{D}C_{m}\right)}$$

The measured count rate can be approximated by:

(2) $$C_{m}=\frac{N_{m}}{t_{i}}$$

where *N*_m_ is the measured number of photon count and *t*_i_ is the integration time for time bins in the time traces. The correct number of photon counts for each time bin of the time traces can then be calculated by:

(3) $$N=N_{m}\frac{1}{1-t_{D}\frac{N_{m}}{t_{i}}}$$

The count rate correction is necessary for DNA fragment sizing based on the fluorescence signal of the individual fragments. As mentioned in the introduction the intercalating dyes used to stain the DNA show a constant ratio of dye molecules per bp, which leads to linear dependence of the fluorescence signal on the fragment length. Without the count rate correction, underestimation of higher count rates would lead to under estimation of fragment lengths especially for larger fragments due to their higher photon count rates [7]. The best measure for the fluorescence intensity of a single DNA fragment is the peak area parameter. It has a better counting statistics than the peak height parameter and for large fragments which can easily extent the size of the focus the peak area is still proportional to the fragment length [5].

### 2.3 Sample preparation

The plasmid DNA used for this study was originally produced as a gene target for *M. tuberculosis* (TB) without the presence of a large more complex genome [13]. This ‘TB Control plasmid’ named pUC19TB consists of a pUC19 plasmid containing an insert including 16S rRNA and rpoB genes from *M. tuberculosis.* The plasmid has a size of 8519 bp. For dPCR the plasmid was linearised and for the flow cytometric detection we used the original form.

The concentration of the pUC19TB plasmid stock solution is too high to be measured directly. Therefore, the material was diluted in three steps with staining in the second step. The dilution was controlled gravimetrically and the final dilution factor was 50725 ± 2191. For staining of the DNA PicoGreen (Molecular Probes Inc., USA) is used as intercalating dye due to its large fluorescence enhancement, wide usable concentration range, and the stability known from literature [14], [15]. The final concentration of PicoGreen in the diluted solution was approximately 0.06 μM. For measurement, the glass syringe was filled with 60 μL of this solution.

## 3 Results and discussion

**Fig. 2:**
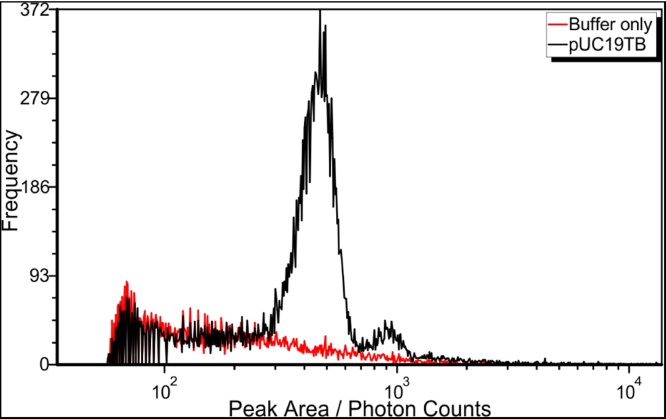
Comparison of peak area histograms of single measurements of dye containing dilution buffer (red) and diluted pUC19TB sample (black). Peak detection for both samples was applied using same settings to get comparable results. The background signal coming from the dilution buffer shows to be stable for both measurements making background subtraction feasible. This holds also for several repeat measurements not shown here.

Fig. 2 shows peak area histograms determined for a background measurement (dilution buffer plus PicoGreen) and the diluted pUC19TB sample. The peak detection algorithm described in materials and methods was applied using same settings for both samples to get analogous histograms. The histogram of the background measurement shows decay of the frequency of events with increasing peak area. When looking at the histogram for the pUC19TB sample the histogram matches the background measurement for peak areas up to 200 photon counts. This match of the two histograms shows the possibility to do a simple subtraction for background correction.

For larger peak areas, the histogram of the pUC19TB sample shows two Gaussian like peaks with mean values of 451 and 922 photon counts. Based on the linear dependence of the peak area on the fragment length or more general on the amount of DNA, the second peak represents signals of approximately double amount of DNA when compared to the first peak. Accordingly, the first peak is attributed to the peak area distribution of single pUC19TB plasmids (8519 bp) and the second one can be attributed to coincidences of two plasmids passing the focal volume or plasmid dimers (17038 bp) due to the nearly perfectly double peak area.

**Fig. 3:**
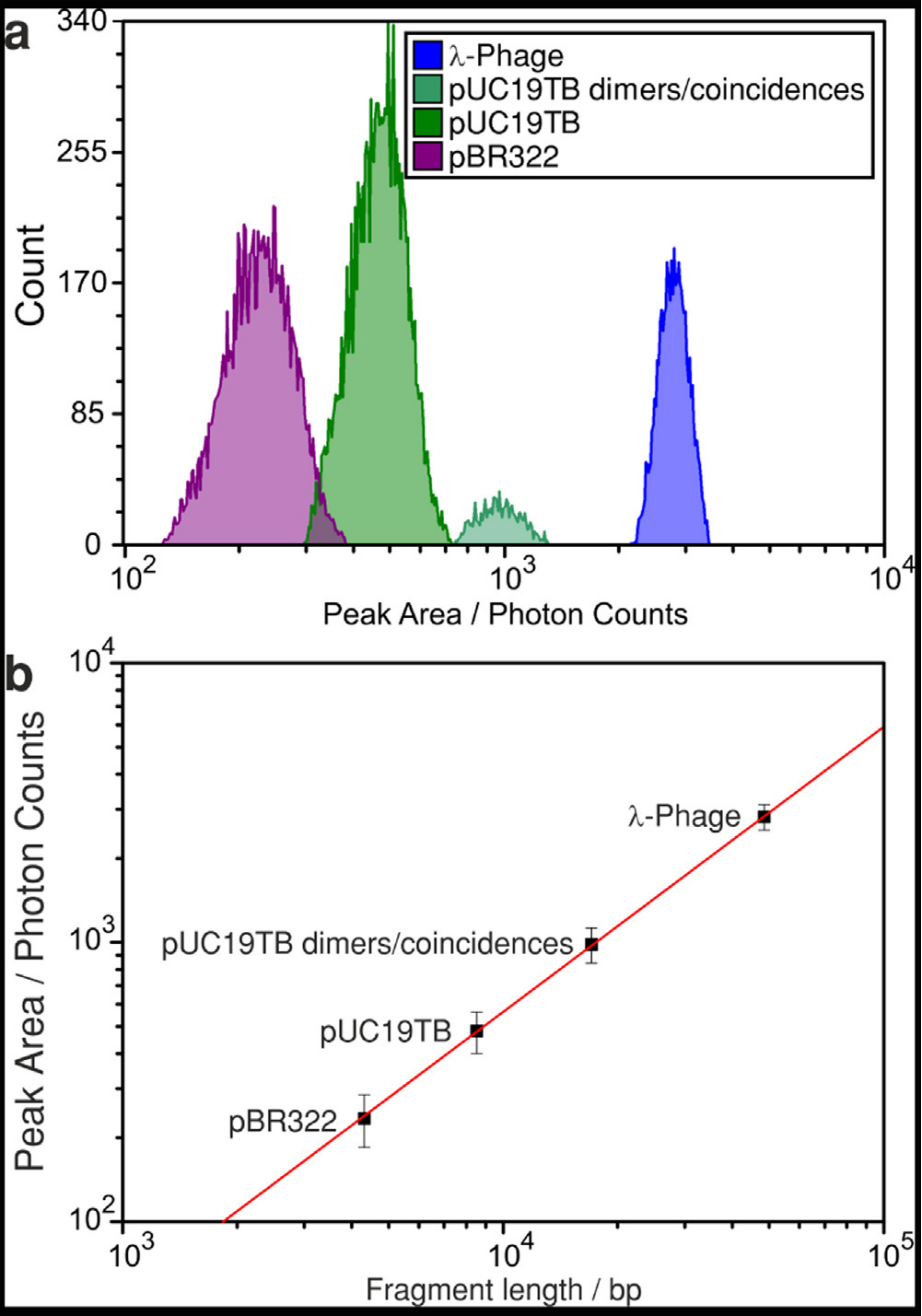
Calibration of the fluorescence intensity of the DNA fragments over fragment length in base pairs (bp). Part a shows the histograms of the distribution of intensities measured for the different samples. Part b shows the resulting calibration curve. The data points and error bars in the graph are derived from Gaussian fits of the histograms in part a, whereby the error bars represent the standard deviation of the fitted gaussians. Linear regression of the data (red line) shows excellent linear relationship of fragment length and fluorescence intensity. The corresponding fragment sizes are 4316 bp, 8519 bp, 17038 bp, and 48502 bp.

To characterise and calibrate this DNA fragment sizing capability of the instrument pBR322 and *λ*-Phage DNA samples with known fragment lengths of 4316 bp and 48502 bp were measured additionally at same laser power as the pUC19TB sample. Fig. 3a shows a compilation of the distributions of measured fluorescence intensities for the pUC19TB sample and for the additionally measured samples. The resulting calibration curve of fluorescence intensity over DNA fragment length is shown in Fig. 3b. The data and error bars for the mean fluorescence intensities of the different types of fragments was derived by Gaussian fits of the distributions of measured fluorescence intensities shown in the histograms in part a of the figure. Linear regression of the data shown as red line points out excellent linear relationship of fluorescence intensity and fragment length on a range of approximately 4000 bp to 50000 bp. To gain such a perfect linear relationship the raw data of the photon counting was corrected for the detector dead time as suggested in ref. [7] using the manufacturer data for the current module to gain true count rates. This compensates for the underestimation of the photon counts for high count rates, which would then result in underestimation of the fluorescence intensity of larger fragments like *λ*-Phage DNA.

The shown excellent sizing performance of the instrument supports the identification of the second peak in Fig. 2. The assumption of detecting pUC19TB dimers or coincidences of two plasmids perfectly fits to the calibration curve. However, looking only at the peak area distribution gives limited insight into effects of coincident events. A better understanding can be derived when looking at the pulse shapes.

**Fig. 4:**
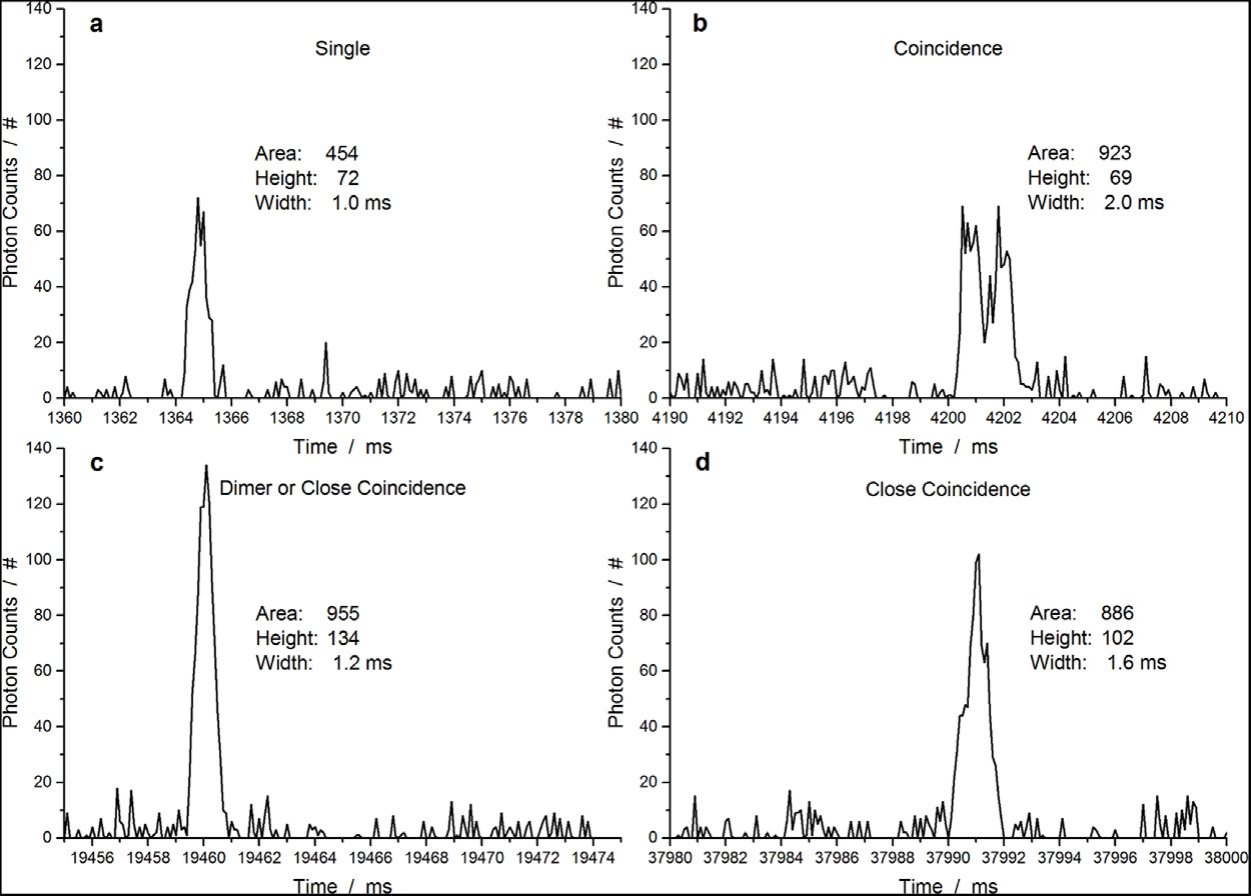
Typical pulse shapes measured for pUC19TB plasmids (Raw data after baseline correction). A single plasmid event is shown in a and coincident detection of two plasmids resulting in a double or broader peak is shown in b and d. Part c shows either a plasmid dimer or perfectly coincident detection of two plasmids, which cannot be distinguished.

Fig. 4 shows typical pulse shapes acquired for the pUC19TB sample and measured peak area, height, and width after base line correction. Part a shows a shape attributed to a single plasmid passing the focus with peak area of 454 photon counts, peak height of 72 photon counts, and peak width of 1 ms. Part b shows a double peak pulse shape caused by coincidence of two plasmids passing the focus closely in time. The peak detection algorithm detects this event as a single peak with peak area of 923 photon counts, peak height 69 photon counts, and peak width 2 ms. Part c shows a peak with area of 955 photon counts, height 134 photon counts, and width 1.2 ms. This peak can be attributed to two individual plasmids passing the focus at the same time or to a plasmid dimer. It is not possible to distinguish these two cases for a single peak. Part c shows a peak with area of 886 photon counts, height 102 photon counts, and width 1.6 ms. Due to the pulse width being significantly longer than for a single plasmid this peak can be attributed to two plasmids passing the focus closely spaced.

Comparing the values determined by the peak detection algorithm it is possible to distinguish following three cases without looking at the pulse shape in detail: Single plasmid, Coincidence of two plasmids, and close coincidence or plasmid dimer. Single plasmids and possible dimers have similar peak width but differ in peak height and peak area by a factor of ∼2. In contrast to that, coincidence events show similar peak area as possible dimers, but the peak width is larger and the peak height is smaller. This means standard flow cytometry software can be used to assign the detected events to the three cases by gating.

**Fig. 5:**
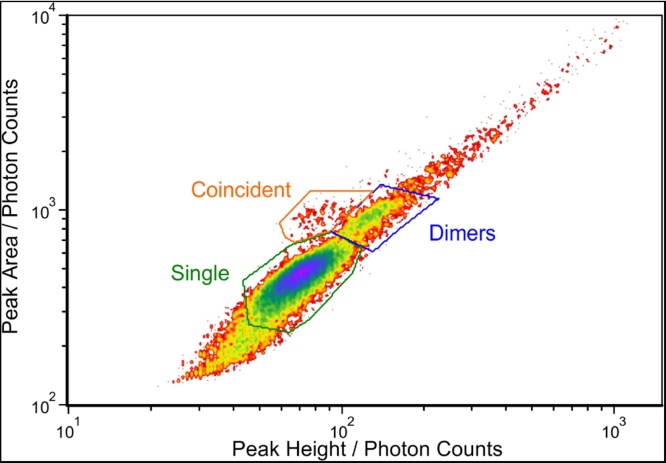
Density plot of peak area versus peak height of the pUC19TB sample. Three gates are shown, which are used to classify events as single plasmids (green), coincidences of two plasmids (orange), and potential dimers (blue).

For visualisation of the data and for setting of the gates the data is plotted in a 2D-density plot of the peak area over peak height as shown in Fig. 5 for the pUC19TB sample. Most of the detected events are on a line from the lower left to the upper right of the diagram. For a single peak pulse shape like for single plasmids or plasmid dimers this fits to similar peak width, because then peak area is proportional to the product of the peak height and the peak width giving a straight line. Therefore, the lower left cluster on this line being the one with most events is identified as single plasmids and the smaller cluster upper right of the first one is identified as possible dimers.

Above the cluster of single events and on the left of the possible dimers a certain number of events is visible. These are attributed to double pulses caused by coincidence of two plasmids because of the reduced peak height in comparison to the possible dimers while showing the approximately double peak area of the single plasmids. This shows the possibility to apply the identification of the different events by just looking at the peak height and peak area, without complex pulse shape analysis.

As mentioned above one drawback of this analysis is the missing possibility to differentiate real plasmid dimers and two plasmids passing the focus at same time. To address this problem the coincidence probability can be calculated based on Poisson statistics to get the expected number of coincidences, which can then be compared to the experimental results. As known for flow cytometric cell counting the total cell count can be approximately corrected for low coincidence probabilities using the mean peak width *τ*, the total cell count *N*_r_, and the measurement duration time *T* [11]:

(4) $$N_{0}=N_{r}\frac{1}{1-N_{r}\tau /T}.$$

The product of the detected events *N*_r_ and the mean peak width *τ*, gives an approximation for the integrated dead time in which no other events can be detected. This can be estimated by the measured integrated dead time *τ*_d_ ≈ *N*_r_*τ* [16], [17], which can be easily calculated from the acquired data for peak width, and hence

(5) $$N_{0}=N_{r}\frac{1}{1-\tau_{d}/T}.$$

The division by the measurement duration time *T* gives the portion of the measurement duration time where no additional events can be detected. For the current data summation of the peak widths of all 22159 detected signals gives an integrated dead time of 19.4309 s. For the measurement duration time of 1200 s this results in a correction of: *N*_0_ = *N*_r_ × 1.0165. This means 1.65% of the detected events are caused by coincident detection of two fragments and the fragment count should be corrected by the factor of 1.0165.

This can now be compared to the fragment counts estimated for pUC19TB using the gating regions shown in Fig. 5. The resulting counts are shown in Table 1 along with the calculated uncertainties for counting statistics, i.e. $u(N)=\sqrt{N}$, rounded as integers. For background correction, same gating regions were also applied to data of a background measurement and the resulting counts were subtracted from the pUC19TB data (see Table 1).

**Table 1:** Resulting DNA fragment counts

	N_sample_	u(N_sample_)	N_backgr._	u(N_backgr._)	N	u(N)	Ratio / %
Single	15264	124	1852	43	13412	131	93.16±0.91
Coincident	154	12	9	3	145	13	1.01±0.09
Dimers	1285	36	445	21	840	42	5.83±0.29

After background correction 14397±138 events were identified as pUC19TB plasmids. Using the correction factor calculated above one would expect 238 (1.65%) to be caused by coincident detection of two plasmids. However, 145 ± 13 (1.01 ± 0.09)% events directly identified as coincidences plus 840 ± 42(5.83 ± 0.29)% events identified as coincidences or plasmid dimers result in 985 ± 44(6.84 ± 0.30)%, which is more than four times larger than expected for statistical coincidences. Taking the expected coincidence rate, this means the number of events identified as coincidences or plasmid dimers is caused by a significant number of plasmid dimers and not only by coincidences. Taking the values in Table 1 together with the expected coincidence rate gives a ratio of (5.19 ± 0.31)% plasmid dimers.

With this analysis, it is possible to determine the concentration of the sample. But, this gives only a first estimate of the concentration. For reliable concentration determination, dilution series measurements have to be performed, which would also improve the analysis of the plasmid dimer rate.

The counts in Table 1 were determined for a sample volume of (1.0086 ± 0.0076) μL. To determine the copy number all events identified as dimers or coincidences give two copies per event and are therefore weighted with a factor of 2. The resulting copy number is (15251 ± 193) copies/μL for the diluted sample and (7.74 ± 0.35) × 10^8^ cp/μL for the stock solution using the dilution factor. For comparison with dPCR results the existence of plasmid dimers is important, because in dPCR dimers cannot be discriminated and would be counted as single plasmids.

However, the impact of plasmid dimers on the dPCR results cannot be validated in the current study, because we used intact plasmids for direct detection and linearised material was used for dPCR (see ref. [13]). For this aspect, we need to use same type of material for both techniques. For stability reasons, it may be better to use intact plasmids for both methods, because intact plasmids especially in supercoiled conformation are known to be very stable [18], which is preferable for reference materials. Additionally, dPCR can be optimised for detection of supercoiled plasmid DNA [18].

As a first attempt for the comparison of both techniques we assume to have no dimers in the material after linearisation. Then in comparison to the concentration of (2.02 ± 0.23) × 10^9^ cp/μL determined by dPCR the result for direct detection is significantly lower (38%). This means less plasmids were counted compared to the dPCR result. Two mechanisms can obviously cause such loss. One possibility is the loss of events because of fragments passing the flow cell outside of the laser focus. This would lead to an asymmetric distribution in the peak area histogram and also to a cut off at smaller peak areas, which can be excluded here.

The second possibility is adsorption of DNA fragments on surfaces of the sample injection system or during sample preparation. In the current setup, most components are typically used for cell experiments and thereby not all surfaces which the sample is getting in contact to are optimised for minimal DNA adsorption making this a probable reason for loss of DNA fragments. For instance, connectors and the cleaving of the capillary reaching into the syringe may adsorb reasonable amounts of DNA.

A strong indication of the adsorption of DNA fragments in the sample delivery system is given by our observation of increasing fragment count rates over time during successive measurements with constant sample flow rate. These were ranging from ∼11.1 nL^−1^ reaching a plateau after approximately 45 min at ∼16.6 nL^−1^ (data not shown). This dynamics is typical for adsorption processes reaching an equilibrium after a certain time making the adsorption of DNA fragments the most probable reason for lower quantification results. Replacing connectors and the capillary by optimised components should help to overcome this issue. However, further investigations including optimisation of sample preparation and application of dilution series experiments is necessary to get reliable results of DNA fragment concentrations in follow-up studies.
